# Chronic Cellulitis in Elephantiasis: A Rare Debilitating Phenomenon

**DOI:** 10.7759/cureus.65855

**Published:** 2024-07-31

**Authors:** Shailja Singh, Rushikesh K Shukla, Akhilesh Singh, Sourya Acharya

**Affiliations:** 1 Medicine, Jawaharlal Nehru Medical College, Datta Meghe Institute of Higher Education and Research, Wardha, IND; 2 Surgery, Jawaharlal Nehru Medical College, Datta Meghe Institute of Higher Education and Research, Wardha, IND; 3 Emergency medicine, Jawaharlal Nehru Medical College, Datta Meghe Institute of Higher Education and Research, Wardha, IND

**Keywords:** leg edema, lymphatic system, chronic cellulitis, lower extremity cellulitis, elephantiasis

## Abstract

Chronic edema, which has multiple etiologies, is predicted to be a significant underlying cause of lymphedema, potentially leading to serious complications. Elephantiasis, characterized by massive swelling of any body part, is a rare but debilitating condition often associated with lymphatic obstruction or anomalies in the lymphatic system. Lymphedema can predispose a patient to cellulitis, an infectious condition with multiple risk factors. This case study presents a 45-year-old male with a history of chronic lymphatic obstruction due to elephantiasis and recurrent cellulitis in his lower limb. Despite receiving multiple courses of antibiotics, the patient continued to experience multiple episodes of cellulitis, along with worsening lymphedema and functional impairment of the limb. The mainstay of treatment for this condition includes compression stockings and surgery, but addressing the root cause of the disease is crucial. Typically, a multidisciplinary approach is required, involving antibiotics, lymph drainage, and compression therapy. This case highlights the challenges faced in managing elephantiasis and its related complications and emphasizes the need for preventive strategies.

## Introduction

Chronic edema, defined as edema lasting more than three months, can have disastrous consequences if not addressed early in its progression. Chronic inflammation of the edematous area causes gradual fibrosis, extracellular matrix remodeling, and adipose tissue deposition over time, all contributing to an irreversible increase in volume of the affected region [1]. Chronic lymphedema is presumed to be the cause of elephantiasis. These individuals often present with significantly swollen and disfigured extremities with thickened, fibrotic-appearing skin, depending on the severity and duration of their condition. Etiologies for lymphedema are classified into primary and secondary. While primary lymphedema can result from blockage, congenital agenesis, or hypoplasia of lymphatic vessels, secondary lymphedema is caused by abnormalities or obstructions of previously existing lymphatic vessels. Worldwide, the most common cause of secondary lymphedema is filariasis. In the West, causes include obesity, radiation therapy, trauma, cancer, recurrent streptococcal lymphangitis, erysipelas, chronic venous stasis, and scleroderma [2]. A rare condition known as elephantiasis nostras verrucosa (ENV) develops when persistent non-filarial lymphedema, caused by bacterial or non-infectious lymphatic blockage, occurs. Patients afflicted with chronic lymphedema are hypothesized to develop a state of chronic inflammation with an accumulation of keratinocytes, adipocytes, and fibroblasts due to the long-term accumulation of protein-rich fluid in the interstitium. This changes the initial soft and swollen tissue into hard, fibrotic tissue with thickened skin [3]. Radiological imaging is rarely necessary as the diagnosis may be based solely on the patient’s medical history and physical examination. However, a biopsy is necessary due to the possibility of an associated malignancy [4,5].

Recurrence of cellulitis is prevalent even with effective treatment. Recurrent cellulitis is linked to substantial medical expenses and potential short- and long-term morbidity. Identifying and managing risk factors, as well as implementing prophylactic interventions such as antibiotic prophylaxis, are necessary to reduce the chance of recurrence [6]. Key risk factors linked to multiple bouts of cellulitis include localized ailments such as venous insufficiency, dermatomycosis, and persistent edema [7]. Pain, swelling, erythema, and warmth are the typical local indications of cellulitis, which can also be linked to a systemic inflammatory response. Lymphatic dysfunction is most likely the primary disease associated with recurrent cellulitis. Lymphoscintigraphy scans of 77% to 87% of individuals with lower limb cellulitis, conducted four weeks following recovery, showed lymphatic abnormalities. Recurrent cellulitis can also be predisposed by any other illness that affects lymphatic drainage and function. Additionally, immunodeficiency and malignancy are systemic diseases that might raise the risk of cellulitis, with a higher probability seen when skin pathology is linked to long-term illnesses like diabetes [6]. Even though cellulitis is commonly seen in community settings, there is no evidence that the techniques developed and tested in secondary care have the same benefit when provided in the community setting [8]. This case study further strengthens the link between cellulitis and chronic lymphatic obstruction.

## Case presentation

A 45-year-old male patient presented with a history of elephantiasis in the right lower limb, spanning over five years, with no prior treatment. Three days prior to presentation, the patient sustained a fall from a bicycle, resulting in trauma to his right foot. Subsequently, he noted an increase in redness and swelling of the affected foot, which was insidious in onset and progressively worsened. Concurrently, he developed respiratory distress, increased drowsiness, and lethargy, and experienced a persistent low-grade fever two days earlier. Initially, the patient was treated at an external medical facility, where he underwent local debridement of the affected site on July 13, 2023. Due to the exacerbation of symptoms, including increased drowsiness and hypotension, along with plans for plastic surgery, he was then referred to our institution for further management. The patient does not have any systemic diseases or illnesses. Notable findings upon examination included a wound measuring approximately 10x6 cm on the lateral aspect of the right lower limb, 5x4 cm on the foot, and 10x7 cm on the medial aspect. Local examination showed that slough was present, indicating tissue necrosis, along with tenderness, while redness was notably absent. Non-pitting edema was also present. A surgical consultation confirmed the diagnosis of cellulitis in the right lower limb, necessitating debridement surgery.

**Figure 1:**
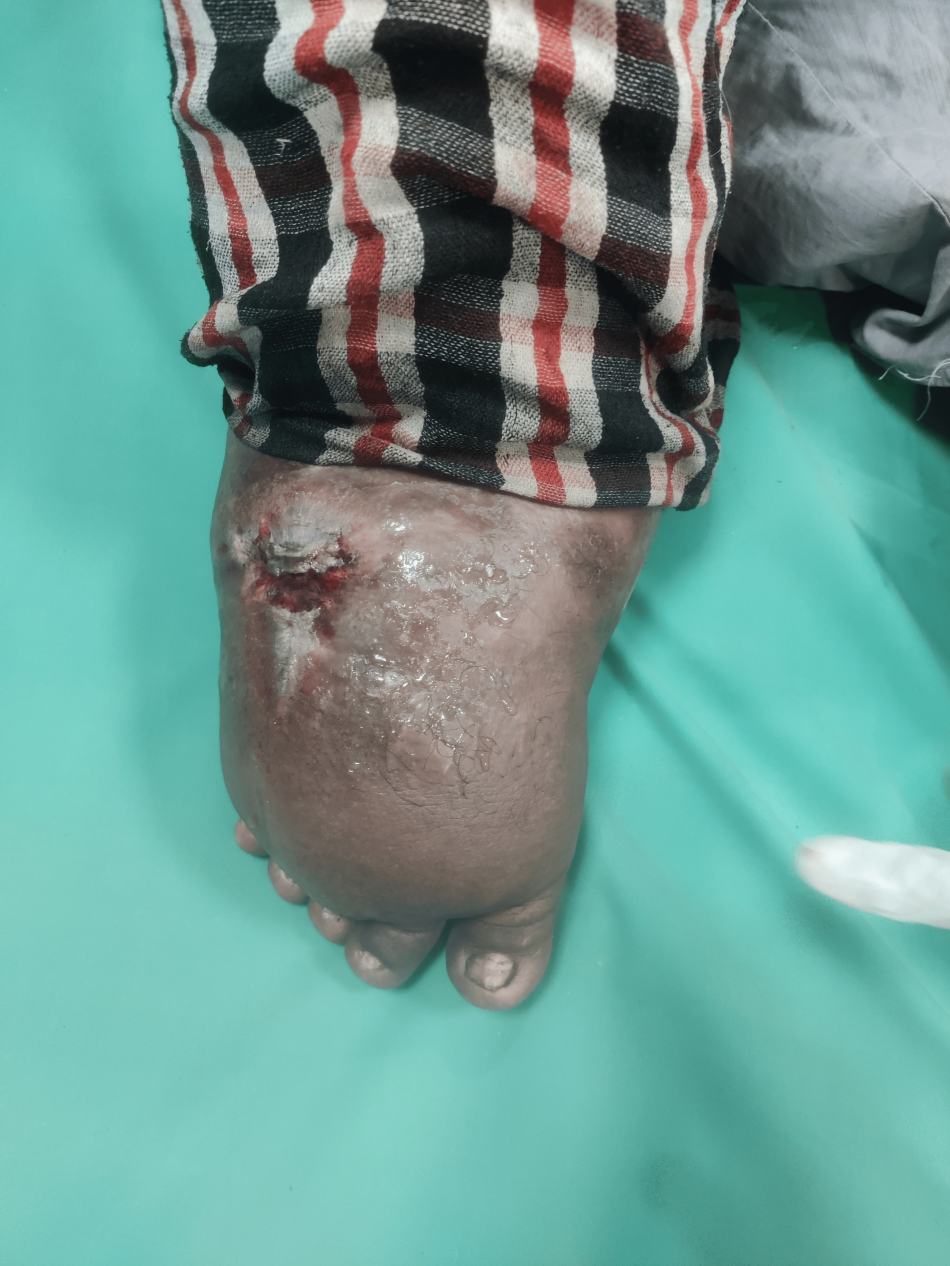
Right foot of the patient showing massive enlargement with a wound on the dorsal aspect.

Figure 1 shows the foot of the patient with massive enlargement (elephantiasis).

**Figure 2:**
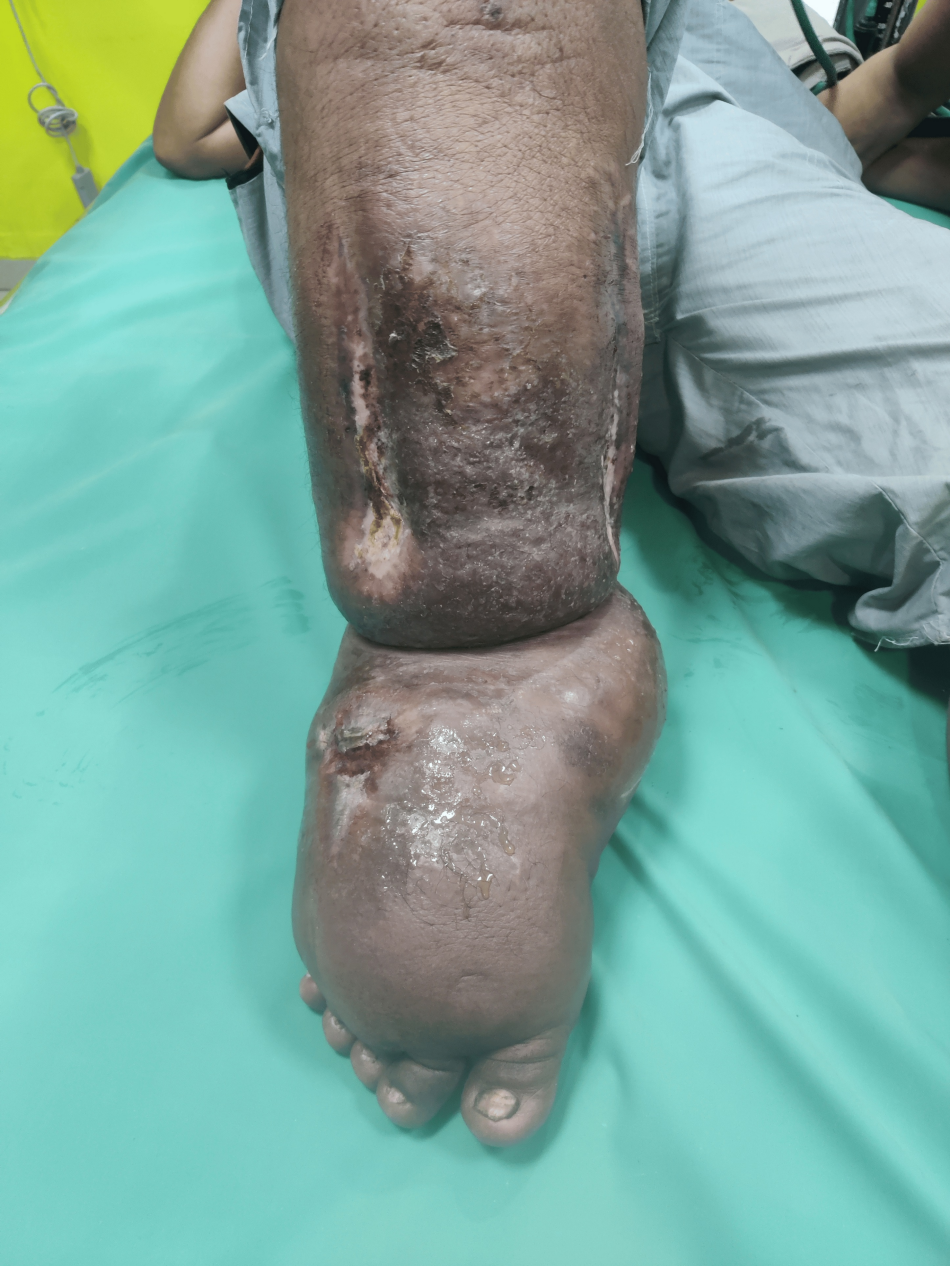
Foot and leg of the patient with multiple wounds.

Figure 2 shows the foot and leg of the patient with multiple wounds.

**Figure 3:**
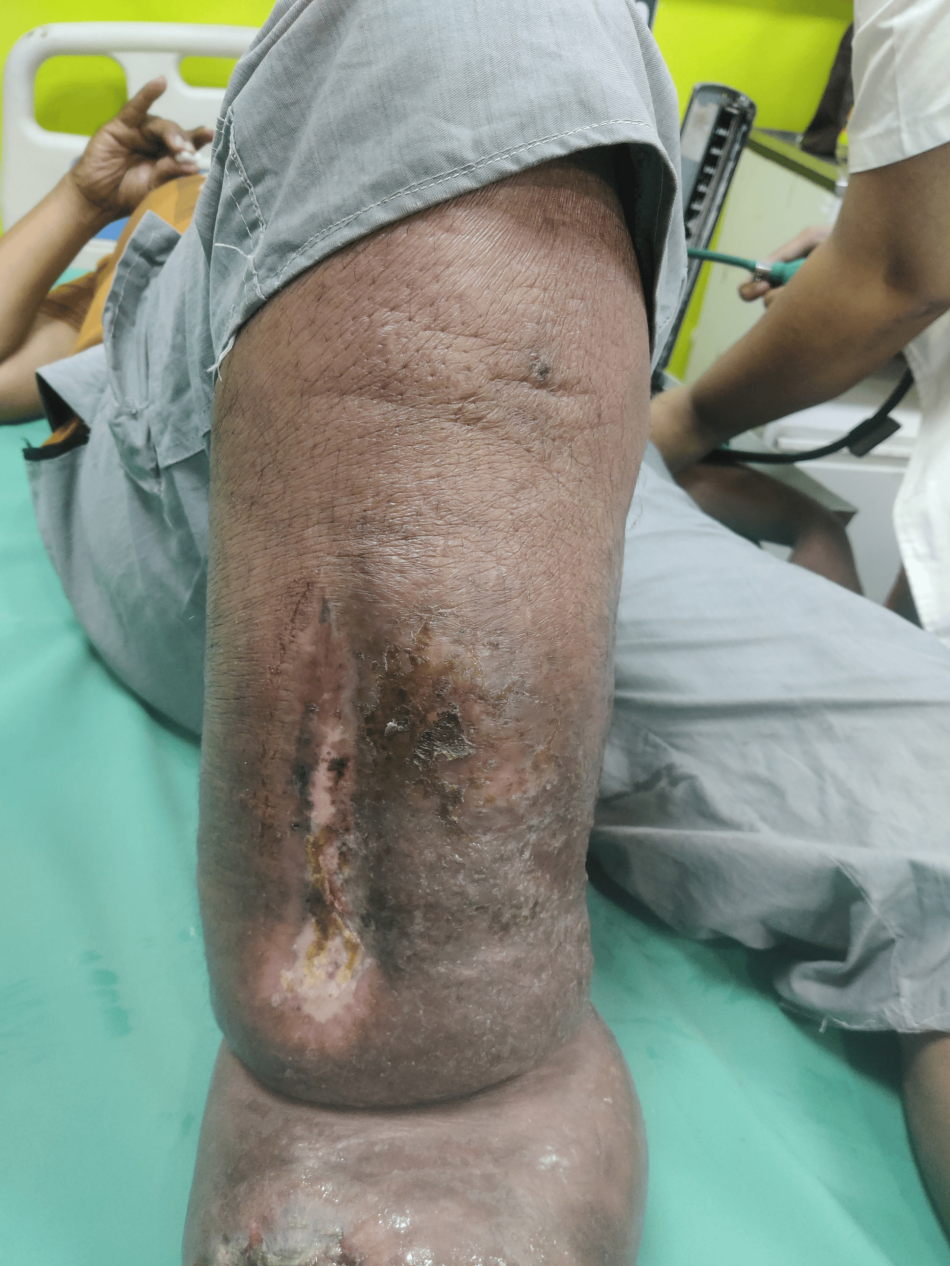
Lateral aspect of the leg.

Figure 3 shows the lateral aspect of the leg with a 10x6 cm wound.

**Figure 4:**
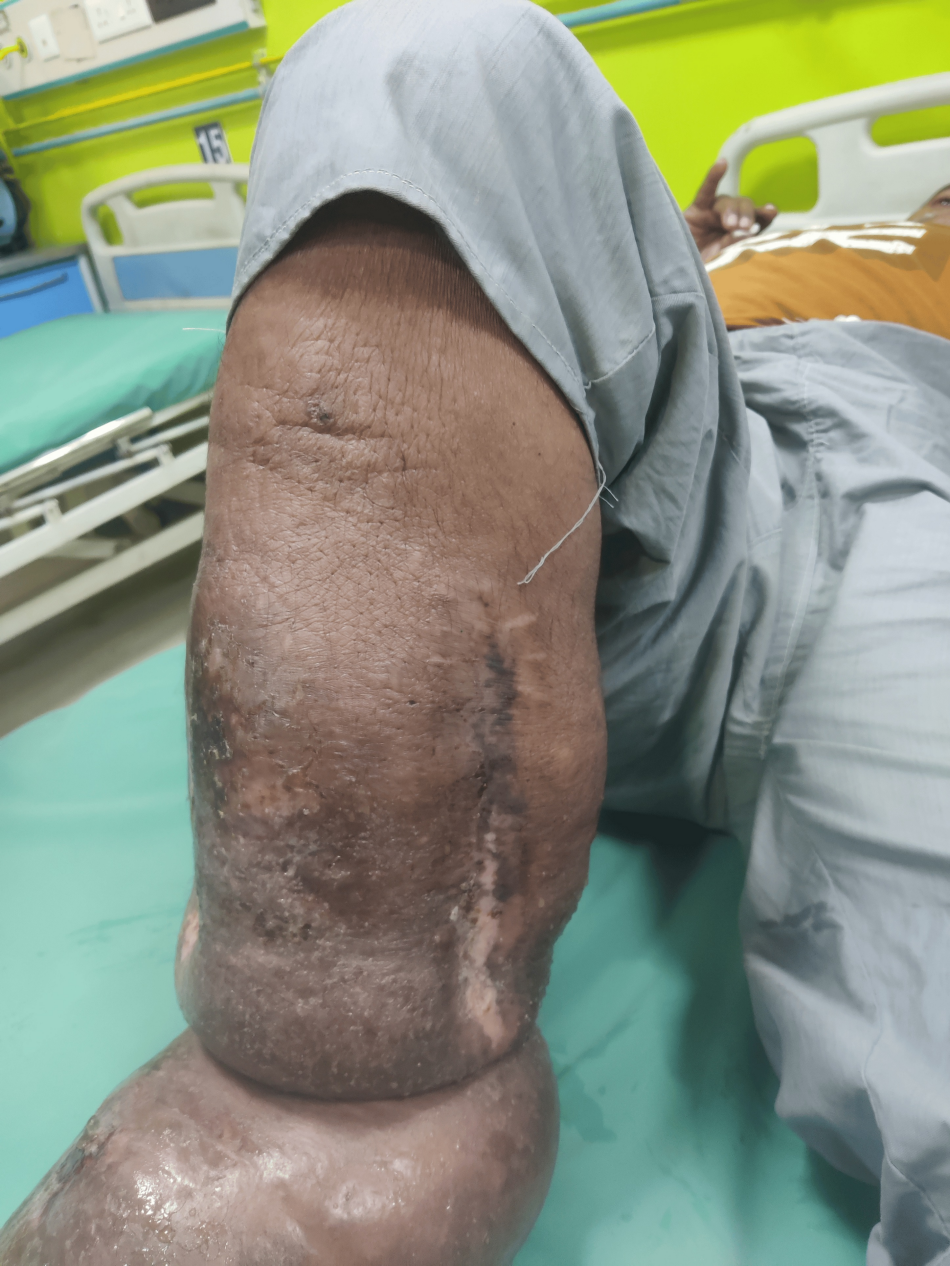
Medial aspect of patient's leg.

Figure 4 shows the medial aspect of the patient's leg with a 10x7 cm wound.

Investigations performed on the patient, mentioned in Tables 1-2, show a microbiology report significant for various bacterial growths. Table 3 shows the antibiotic sensitivity report for the mentioned organisms.

**Table 1:** Blood investigations. APC: Absolute Platelet Count.

RBCs	Normocytic normochromic
WBCs	14,700 – Total count increased on smear with neutrophilic leukocytosis and a mild shift to the left up to band form; a few show toxic granules and cytoplasmic vacuolations; platelets - reduced on smear.
APC	93,000 cells/mm³ as per cell counter. No hemoparasites seen.
Metamyelocytes	12%
Neutrophils	8%
Band forms	60%
Lymphocytes	15%
Monocytes	3%
Eosinophils	2%
Basophils	0%

**Table 2:** Microbiology report of patient's wound sample. HPF: High power field.

Tissue from cellulitis	Sample from debrided wound
Growth of Klebsiella species	Growth of Citrobacter koseri species
Growth of Acinetobacter species	Growth of Pseudomonas aeruginosa species
1-2/hpf pus cells seen / 1-2/hpf pus cells seen	1-2/hpf pus cells seen
Gram-negative bacilli seen	Gram-negative bacilli seen

**Table 3:** Antibiotic sensitivity report.

Organism	Amikacin	Amoxiclav	Ampicillin	Cefipime	Cefotaxime	Ceftazidime	Ceftriaxone	Ciprofloxacin	Colistin
Acineobacter species	Resistant			Resistant		Resistant	Resistant	Resistant	Sensitive
Klebsiella species		Resistant	Resistant	Resistant	Resistant			Sensitive	Sensitive
Citrobacter species			Resistant		Intermediate		Sensitive	Resistant	Sensitive
Pseudomonas aeruginosa				Resistant		Resistant		Resistant	

Daily dressing and wound care were provided. The following drugs, mentioned in Table 4, were administered to the patient during the course of his hospital stay.

**Table 4:** Drugs given to the patient during his hospital stay.

Drug	Duration
Injection Piperacillin/Tazobactam 2.25 gm intravenously three times daily (TDS)	14 days
Injection Clindamycin 600 mg intravenously twice daily (BD)	14 days
Injection Levofloxacin 500 mg intravenously once daily (OD)	14 days
Injection Pantoprazole 40 mg intravenously once daily (OD)	
Injection Diclofenac Sodium (50.0 mg) + Paracetamol (325.0 mg) intravenously twice daily (BD)	
Injection Paracetamol intravenously as needed (SOS)	
Enterogermina respules three times daily (TDS)	
Syrup Sucralfate (500.0 mg) + Oxetacaine (10.0 mg) 2 teaspoons three times daily (TDS)	

The following drugs mentioned in Table 5 were prescribed to the patient on discharge.

**Table 5:** Drugs given to the patient on discharge.

Drug	Duration
Tablet Methylcobalamin 1500 mg once daily (OD)	15 days
Tablet Pantoprazole once daily (OD)	7 days
Tablet Tramadol 37.5 mg and Acetaminophen 325 mg twice daily (BD)	7 days
Tablet Paracetamol 650 mg as needed (SOS)	7 days
Syrup Sucralfate (500.0 mg) and Oxetacaine (10.0 mg) 2 teaspoons three times daily (TDS)	7 days

The patient was also advised to maintain daily sterile dressing and limb elevation. The patient was told to return for a review in the OPD after seven days.

Three months later, the patient presented to the hospital with similar complaints. A wound swab was sent for culture, and the report was significant for growth of Pseudomonas aeruginosa and Klebsiella pneumoniae. The patient was managed with daily dressing, debridement, and local spray of Inj amikacin; the patient underwent three platelet-rich plasma injection sittings and one vacuum-assisted closure application for seven days.

A USG color Doppler of the right lower limb was performed and noted significant findings: There was no evidence of an echogenic focus in the common femoral vein, superficial femoral vein, popliteal vein, anterior tibial vein, or posterior tibial vein of the right lower limb. No evidence of deep vein thrombosis was seen. Good flow was noted in the common femoral artery, superficial femoral artery, and popliteal artery with triphasic forward flow. The anterior tibial artery, posterior tibial artery, and dorsalis pedis artery showed biphasic flow, possibly due to edema.

During the course of the patient's hospital stay, he was administered the following drugs mentioned in Table 6, along with advice for limb elevation. The patient was then discharged per his request.

**Table 6:** Drugs prescribed to the patient on his second hospital visit.

Drug	Route	Frequency
Injection Piperacillin/Tazobactam 2.25 gm	IV	Three times daily (TDS)
Injection Metronidazole 100 ml	IV	Three times daily (TDS)
Injection Tramadol 2 ml in 100 ml normal saline	IV	Three times daily (TDS)
Tablet Pantoprazole 40 mg	IV	Once daily (OD)
Tablet Ondansetron 4 mg	IV	Three times daily (TDS)
Tablet Diethylcarbamazine 100 mg	Oral	Three times daily (TDS)
Tablet Coumarin 200 mg	Oral	Three times daily (TDS)
Tablet Sodium Bicarbonate 500 mg	Oral	Three times daily (TDS)
Tablet Elemental Calcium 500 mg + Vitamin D3 250 IU	Oral	Twice daily (BD)

## Discussion

Unfortunately, lymphedema is a prevalent condition whose incidence is frequently underreported. According to the International Society of Lymphology, a severe form of chronic lymphedema advancement is lymphostatic elephantiasis, which divides lymphedema progression into four phases (0-3) [9]. Edema from any source that persists for over three months is now considered chronic edema. Chronic edema compromises tissue viability, reducing oxygenation and nutrients to the cells. It may also result in persistent inflammation and cellular debris buildup, leading to fibrosis and lymphatic dysfunction, thereby increasing the risk of infection and ulcers. Chronic edema is primarily caused by obesity, lymphedema, venous insufficiency, and immobility, often with multifactorial causes. Lymphedema should not be ruled out even in the absence of typical symptoms, particularly when the edema is non-pitting and not relieved by elevation. The presence of lymphedema in patients with cellulitis may be linked to a prolonged illness period and fever. Recurrent cellulitis is predicted to develop in more than one-third of individuals with chronic edema, and the risk rises with edema severity [7]. ENV, which is a possible diagnosis here, has differential diagnoses including papillomatosis cutis carcinoides, lipedema, lymphatic filariasis, lipodermatosclerosis, pretibial myxedema, chromoblastomycosis, and Stewart Treves syndrome. Characteristic histological findings of ENV include pseudoepitheliomatous hyperplasia, dilated lymphatic channels, enlarged tissue gaps, and widespread fibrous tissue hyperplasia within the dermis, subcutaneous tissue, and lymphatic artery walls [10,11].

Treatment

Although lymphedema is widespread and debilitating, individuals typically receive inadequate and substandard therapy. Graduated compression stockings (GCS) are widely used to treat lymphedema and chronic edema caused by chronic venous disease. These stockings apply the highest pressure at the ankle, and the compression intensity decreases proximally. Class 1 compression is defined as pressure less than 20 mm Hg; class 2 is pressure between 20-30 mm Hg; and class 3 is pressure of 30 mm Hg or more. Patients with peripheral neuropathy, peripheral vascular disease, or sensitivities to stocking materials should avoid GCS. Hyperbaric oxygen therapy has also been approved as an adjunctive therapy for wound healing. It works through two mechanisms: primary hyper-oxygenation and secondary mechanisms including vasoconstriction, angiogenesis, and antibiotic synergy. Prophylactic antibiotic treatment is now limited to non-purulent illnesses and is designed to prevent infections by beta-hemolytic streptococci, notably Streptococcus pyogenes [7]. Surgery for lymphedema is typically divided into two categories: physiological surgery, aiming to restore lymphatic flow, and reductive surgery, which removes accumulated subcutaneous fibroadipose tissue. These surgical alternatives are often used in conjunction with conservative therapy approaches. Advances in microsurgical techniques and a better understanding of lymphatic anatomy have made such physiological operations possible. Procedures like lymphaticovenular anastomosis (LVA) and vascularized lymph node transfer (VLNT) allow patients to improve lymphatic function and address the underlying causes of lymphedema. Moreover, more precise preoperative planning enhances the likelihood of success, thanks to contemporary imaging techniques such as lymphoscintigraphy, ultra-high frequency ultrasound, indocyanine green angiography, and near-infrared fluorescence imaging [1]. Decongestive lymphatic therapy is a comprehensive form of physical treatment that includes skin cleanliness, limb compression, and exercise [12]. Additionally, reports of success with external sequential pneumatic compression devices exist. It is notable that liposuction has been recommended for more straightforward cases. Oral or topical retinoids have been successfully used in pharmacological intervention and may be used as a supplement to physical therapy [12].

Surgical techniques should be tried in instances that have not improved with conservative or medicinal treatment. Numerous case studies have documented the effective reduction of verrucous lesions with surgical debridement. While surgical debulking eliminates extraneous skin and subcutaneous tissue, it does not treat the underlying lymphatic disease. Despite this, patients experience more comfort. Numerous case studies have reported successful lymphedema treatments using different microsurgical techniques. Both LVA and free muscle flap transfer have been employed, albeit infrequently, to treat obstructive lymphedema in individuals. Additional therapies like deodorant powders and topical keratolytics (such as salicylic acid) are frequently employed to control odor. Limb amputation may be considered for cases which are not responding to treatment [13]. LVA is a helpful surgical procedure for treating secondary lymphedema in the lower limbs. Furthermore, subsequent lymphedema lesions such as acquired lymphangioma circumscriptum and ENV respond well to this surgical treatment [14]. In research by Demirtas Y et al., 42 individuals with unilateral lymphedema of the lower extremities underwent microlymphatic surgery. Surgery outcomes were categorized as good, mediocre, or ineffective based on clinical evaluations using lymphoscintigraphy and volume measurements. After 11 months of follow-up, the average edema volume had decreased by 59.3%. There were 28 good outcomes, 8 mediocre outcomes, and 6 ineffective outcomes categorized. In certain cases, supermicrosurgical LVA and/or lymphaticovenous implantation may be recommended as the preferred course of treatment since they appear to be quite helpful, particularly in the early stages of peripheral lymphedema [15].

## Conclusions

To sum up, persistent lymphatic blockage and stasis pose a serious problem for patients, resulting in debilitating diseases like elephantiasis and recurrent cellulitis, which, if not curable, are at least manageable. The sequence of events from the first set of symptoms to irreversible tissue alterations highlights the significance of prompt intervention and treatment. The complex link between lymphatic dysfunction and recurring infections is shown by the high prevalence of lymphatic anomalies in individuals affected by and recovering from cellulitis. A comprehensive strategy is needed to address chronic edema, one that includes managing underlying causes, implementing prevention measures, and possibly providing antimicrobial prophylaxis to high-risk individuals. Optimizing patient outcomes and reducing the burden of recurrent cellulitis and its related morbidity and healthcare costs require developing efficient tools and interventions suited for community settings. For this reason, further research and proper guidelines are crucial to manage this condition. These developments represent a significant improvement in the treatment of this crippling illness, which lowers healthcare costs and improves patient outcomes.
